# C_60_ Fullerene as an On-Demand Single Photon Source at Room Temperature

**DOI:** 10.1021/acs.nanolett.5c04007

**Published:** 2025-10-03

**Authors:** Raul Lahoz Sanz, Lidia Lozano Martín, Adrià Brú i Cortés, Sergi Hernández Márquez, Martí Duocastella, Jose M. Gómez Cama, Bruno Juliá-Díaz

**Affiliations:** † Departament de Física Quàntica i Astrofísica, Facultat de Física, 223309 Universitat de Barcelona (QCommsUB group), 08028 Barcelona, Spain; ‡ 553397 Institut de Ciències del Cosmos (ICCUB), Universitat de Barcelona (UB), c. Martí i Franqués, 1, 08028 Barcelona, Spain; ¶ Departament d’Enginyeria Electrònica i Biomèdica, Universitat de Barcelona (UB), c. Martí i Franqués, 1, 08028 Barcelona, Spain; § Institute of Nanoscience and Nanotechnology (IN2UB), Universitat de Barcelona (UB), 08028 Barcelona, Spain; ∥ Department of Applied Physics, Universitat de Barcelona, C/Martí i Franquès 1, 08028 Barcelona, Spain; ⊥ Institut d’Estudis Espacials de Catalunya (IEEC), Edifici RDIT, Campus UPC, 08860 Castelldefels, Barcelona, Spain

**Keywords:** Single-photon source, fullerene, quantum communications, quantum technologies, quantum light.

## Abstract

Single photon sources are fundamental for applications in quantum computing, secure communication, and sensing, as they enable the generation of individual photons and ensure strict control over photon number statistics. However, current single photon sources can be limited by a lack of robustness, difficulty of integration into existing optical or electronic devices, and high cost. In this study, we present the use of off-the-shelf C_60_ fullerene molecules embedded in polystyrene as room-temperature reliable single-photon emitters. As our results demonstrate, these molecules exhibit on-demand single-photon emission, with short fluorescence lifetimes and, consequently, high emission rates. The wide availability and ease of preparation and manipulation of fullerenes as single photon sources can pave the way for the development of practical, economic and scalable quantum photonic technologies.

## Graphical abstract

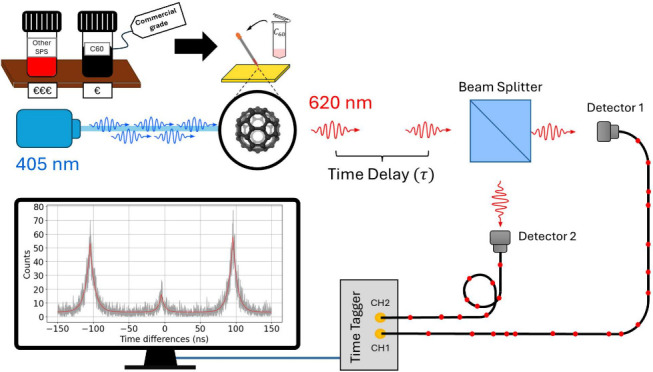

Single photon sources (SPSs) have become a cornerstone in quantum technology applications in sensing,^1^ computing^2,3^ and communications. They are crucial for quantum key distribution (QKD),^4,5^ where encoding information in a single photon helps prevent potential photon number splitting attacks.^6^ The growing number of applications require the development of optimal sources of individual photons. These should ideally be bright, stable, and easy to fabricate and operate.

Brightness - the ability to emit a high number of photons per unit time - is related to the radiative excitonic state’s decay time, and dictates the speed at which information can be exchanged. Stability can be compromised by the intermittency of the emission, referred to as blinking, or by a gradual decline of the emission over time, known as bleaching. A low production cost combined with room temperature operation is essential for the transition toward large-scale implementation. Additionally, there is a need for on-demand single-photon generation, that is, the possibility to trigger emission using an external and controllable signal. To this end, the source must rely on discrete energy levels, as in quantum dots or atomic systems, where strong carrier confinement leads to quantized energy levels. The distribution of these levels is also relevant. For instance, fast, nonradiative, Auger recombination can help suppress multiexciton emission,^7^ increasing the likelihood that a single photon is emitted per excitation cycle.

Recent progress has brought the concept of an ideal SPS closer to reality. However, the commonly used sources—epitaxial quantum dots,^8^ colloidal quantum dots (CQD), and nitrogen-vacancy (NV) centers in nanodiamonds^9,10^ —still present significant trade-offs. Epitaxially grown quantum dots offer high brightness and excellent emission stability,^11,12^ but their production cost is high and require cryogenic temperatures during operation.^13^ CQDs, in contrast to epitaxial ones, exhibit strong quantum confinement, keeping carriers trapped in discrete energy levels and preserving single-photon purity even at room temperature. They are inexpensive to produce, but typically exhibit exciton lifetimes of 20 ns when not coupled to optical cavities.^14,15^ These sources suffer from emission intermittency^16^ and short operational lifetimes, often limited to a few minutes, which ultimately restricts their brightness.^17^ An alternative that addresses the issue of emission intermittency is the use of NV-centers in nanodiamonds.^18–20^ In these systems, blinking is almost completely suppressed, and in some cases, stable emission lasts several hours. However, their brightness is typically low, as only a small fraction of the emitted photons can escape the nanodiamond and reach the far field. Their broadband spectra can also be a drawback, e.g., for applications requiring monochromatic light or when coupling to resonant cavities is needed.

Carbon-based SPSs, e.g. graphene quantum dots^21^ and carbon nanotubes,^22,23^ have emerged as a new class of SPSs. Their emission properties can be tuned, via edge functionalization in graphene, and by varying their diameter in nanotubes,^24^ enabling the engineering of their optical properties. In addition, they can operate at room temperature.

Alternatively, it is possible to use molecules as sources of single-photon emission. Examples range from optical excitation in Oxazine 720 ^25^ or terrylene molecules^26,27^ to electrical excitation in ZnPc molecules.^28^ Single molecules present several appealing characteristics for single-photon emission. Their large transition dipole moments and short spontaneous emission lifetimes contribute to high brightness and emission efficiency. However, single molecules also face notable limitations. Issues such as photobleaching and emission intermittency can hinder their long-term operation, while environmental sensitivity introduces instability and reduces the efficiency of single photon emission.^29^

Here, we show how C_60_ fullerene molecules can operate as novel, low-cost, and room-temperature single photon sources. In addition, their significantly shorter radiative lifetime compared to other SPSs, such as CQDs, enables on-demand high emission rates. To the best of our knowledge, the functionality of C_60_ fullerenes as SPSs has only been demonstrated by excitation via charge injection through quantum tunneling from the tip of a scanning tunneling microscope cantilever.^30^ Our approach is more accessible and easier to implement, relying on the optical excitation of off-the-shelf C_60_ fullerene molecules embedded in polystyrene for their preservation. We conduct an extensive study of their emission properties, including antibunching experiments, measurement of the exciton lifetimes, and analysis of their blinking statistics, both under continuous as well as pulsed light excitation.

To prepare our samples, we use dry toluene with 5% (w/w) polystyrene. In this solution, we dissolve 1% by mass of our fullerene sample (*Sigma-Aldrich, 379646*). Then, we begin a serial dilution, obtaining successive dilutions of 1:100 and 1:10000 from the initial solution, where the solvent is our dry toluene with 5% mass of polystyrene. This dilution process is carried out inside a glovebox with an *N*_2_ atmosphere. Next, we deposit a 5 μL drop onto a gold-coated silicon wafer, while the wafer rotates at 4000 rpm inside the spin-coater. At the moment the drop is deposited, we allow it to spin for 1 min to ensure proper spreading of the drop. Once the droplet has spread across the entire gold-coated surface, the rapid evaporation of the toluene leads to the formation of a polystyrene layer in which the fullerene molecules are embedded. Here, the polystyrene film formed during spin coating helps to preserve the sample during a long period of time.^31^

After sample preparation, we use the setup depicted in Figure 1 to excite and collect light from an individual single photon source. For the optical excitation, we use a 405 nm laser (*USB-Powered Laser Module, Flim Laboratories*), that can work both in the continuous and pulsed regime, with a pulse duration of 50 ps. The laser light is focused on the sample with an air-based Nikon objective (0.9 N.A., 100×). This blue light excites our sample, and the emitted light from the source, upon de-excitation, is also collected by the objective and directed to a dichroic mirror (*DMLP567, ThorLabs*) with a cutoff wavelength of 567 nm. Light transmitted through the dichroic mirror, i.e. wavelengths above 567 nm, also pass through a long-pass filter (*FELH0550, ThorLabs*) and a band-pass filter (*MF620–52, ThorLabs*). This ensures that only light with a wavelength around 620 nm is detected, while also preventing any residual blue laser light from contaminating our measurements. Finally, light is sent to a Hanbury Brown and Twiss (HBT) setup for measuring the second-order autocorrelation function, see Figure 3a.

**Figure 1:**
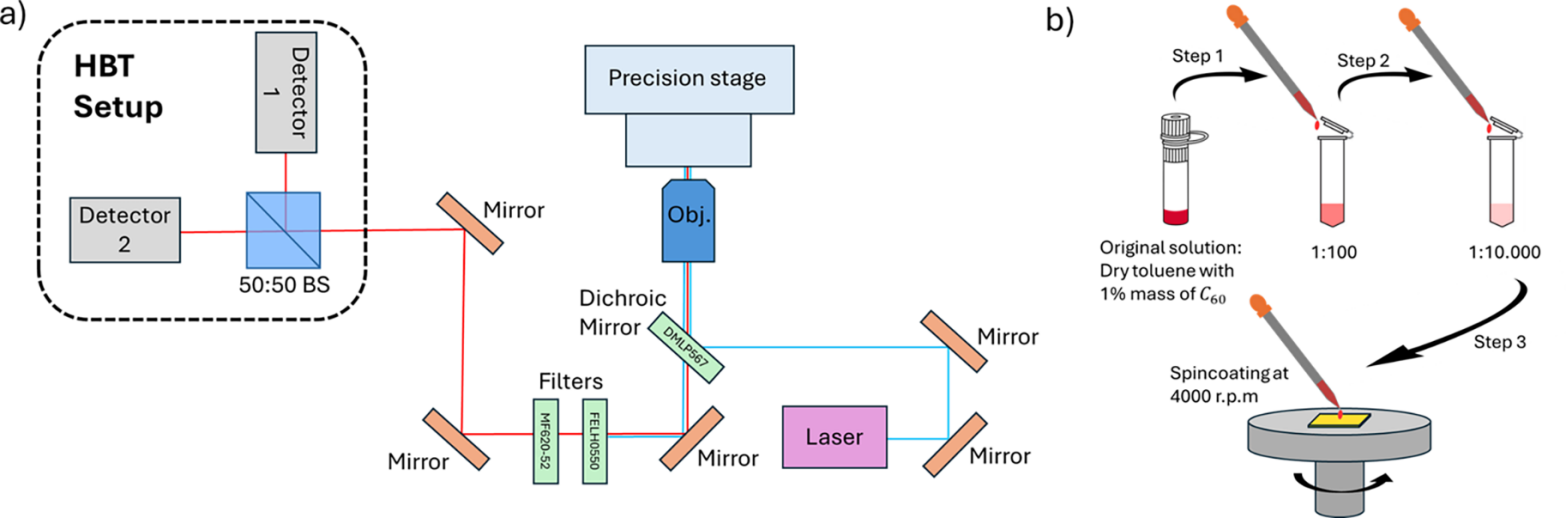
a) Scheme of setup used for the excitation and light collection of an individual single photon source. The Hanbury Brown and Twiss (HBT) setup used for measuring the second-order autocorrelation function is also shown. The 50:50 nonpolarizing beam splitter (50:50 BS) effectively splits the light in two channels. The first one (Ch 1) leads the light to the detector 1, the start detector, while the second one (Ch 2) leads the light to the detector 2, the stop detector. b) Schematic of the sample preparation process. Starting with a dry toluene sample in which 1% by mass of fullerene is dissolved. From this original solution, a serial dilution is performed to obtain two additional samples with concentrations of 1:100 and 1:10000, using dry toluene as the diluent. Finally, a drop is deposited onto a gold-coated substrate while it is spinning at 4000 rpm.

The HBT setup consists of a 50:50 nonpolarizing beam splitter that splits each photon into a superposition of being on each arm of the HBT. Each arm guides the light into a fiber coupler. After coupling into a multimode fiber, the photons are directed to two different channels of our single photon detector (*SPCM-AQ4C, Excelitas*) based on silicon avalanche photodiodes. The electronic pulses produced by the detector upon photon detection are sent to a time-correlated single photon counting (TCSPC) module (*TT-Ultra, Swabian Instruments*). This device records the exact arrival time of each event at each channel, as well as the synchronization pulses from the pulsed laser when it is working in the pulsed wavelength (PW) mode. Since our TCSPC module has a precision of about 50 ps and our single-photon detectors have a precision of around 600 ns, the main source of error in measuring the arrival time of each photon comes from the detectors. In contrast, the measurement of the synchronization signal for each laser pulse is only affected by the TCSPC module’s uncertainty, which is 50 ps.

First, we characterize the emission properties of the C_60_ molecules using a highly concentrated sample (1:100). The emission spectrum of the molecules shows a band centered at 2 eV (620 nm), as seen in the photoluminescence spectra reported in Figure S1. Interestingly, the location of this peak is not consistent with self-trapped polaron emission.^32^ A possible explanation of the observed spectrum is photoinduced emission, in which light emission is enhanced or altered upon exposure to ultraviolet or visible light (see Supporting Information). As shown in Figure S1, a progressive enhancement of emission from our C_60_ molecules is observed upon excitation with 405 nm laser light, consistent with the phenomenon of photoinduced emission. This process can be explained by the photo-oxidation of C_60_ when using photons with energies within the absorption band of the molecule. Thus, the molecules undergo oxidation by reacting with molecular oxygen, resulting in the formation of oxidized fullerene species with lower symmetry, such as C_60_O_n_. The lower symmetry enhances the HOMO–LUMO (highest occupied molecular orbital - lowest unoccupied molecular orbital) transition. This transition was originally forbidden in the highly symmetric pristine C_60_, thereby increasing the photoluminescence emission. This oxidative modification leads to a permanent increase in fluorescence intensity and a spectral blue shift observed during the irradiation process.^33^

Although all evidence points to the emitting species being oxidized fullerene molecules, C_60_O_n_, the exact number of oxygen atoms attached to each fullerene molecule cannot be determined with our current experimental setup. Still, the Raman spectrum of one of our samples features a significant contribution attributed to the oxidation of fullerene molecules, see Figure S2. This indicates that these oxidized species are prevalent in our samples. Therefore, from this point on, whenever we refer to a fullerene molecule behaving as a single photon source, we will be referring to an oxidized fullerene molecule of the type C_60_O_n_.

After demonstrating light emission from C_60_ molecules, we investigate their behavior as single photon emitters. We characterize the temporal emission of a highly diluted sample (1:10000), i.e. the molecules are sparse enough to be analyzed individually. Figure 2a,b show a characteristic PL emission trace from a single C_60_ molecule under CW excitation. Notably, our source features blinking, i.e. fluctuations between two distinct emission rates, which is a strong indication that we are exciting a single emitter. Blinking can be attributed to Auger ionization. In this process, the fullerene molecule becomes temporarily ionized when either an electron or a hole is expelled from the interior (see Figure 2d). During this period, the source does not exhibit radiative emission.^34,35^

**Figure 2:**
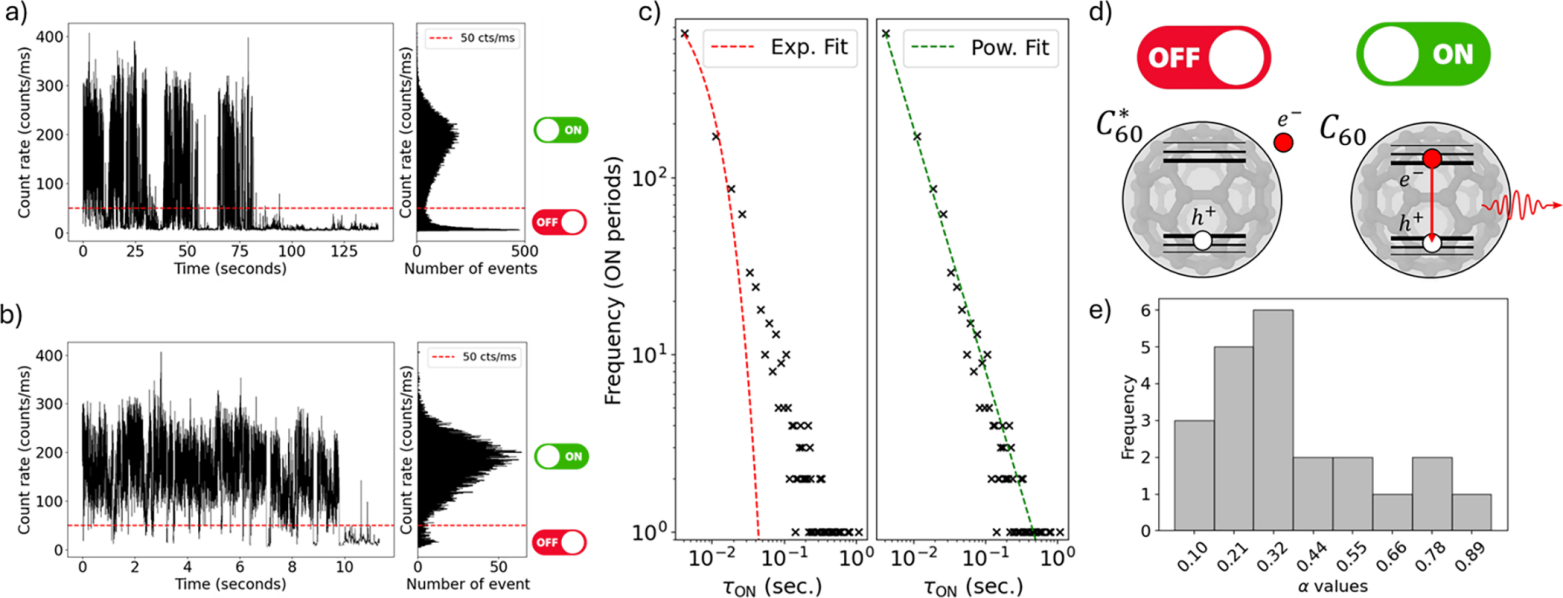
Blinking behavior of our sources. a) PL intensity of the emission during all the measurement. b) Detail of the first 12 s of the PL intensity measurement. In both graphs, the ON and OFF switches represent the periods when our source is emitting or not, respectively, while the threshold separating these two behaviors is set at 50 counts/ms. c) Graph illustrating the fits to an exponential curve (left) and a power-law curve (right) for the duration of the different periods in which our source remained in the ON state (τ_ON_). d) Scheme illustrating the state of our sources during ON and OFF periods. e) Histogram representing the different values obtained of |α| in the power-law fitting in different C_60_ molecules.

Further analysis of the emission statistics of individual C_60_ molecules can reveal more details about their photophysical properties. The distribution of counts features a marked bimodal distribution (see Figure 2a,b). This can be attributed to the constant switching between two different states, i.e. ON and OFF. In our analysis, we fixed a threshold of 50 counts per millisecond to separate the ON and OFF states. The average count rate detected for the ON state is around 200 counts/ms, while for the OFF state is around 20 counts/ms, above the dark count rate from our detectors of 1 count/ms. The difference between the count rate for the OFF state and the expected count rate due to the dark counts may be due to the fluorescence of the sample and the background noise of the laboratory. The distribution of the duration of the ON periods, τ_ON_, can be related to the three- or multistate nature of the photon emission,.^34,35^ The three-level description predicts an exponential decay of the distribution.^34^ Our data does not follow an exponential decay. Instead, we observe a clear power-law behavior ∝ τ_ON_^–(1+α)^, see Figure 2 c), indicating that the emission of individual photons requires a more complex model.^35^ Note that the particular molecule analyzed in the figure exhibits a value of α = 0.369 ± 0.011. Repeating the measurement, a distribution of α values is obtained, see Figure 2e). All values fall within the interval 0 < α < 1.^36^ This type of intermittency behavior in the emission of single photon sources has already been observed in different types of emitters, such as CQDs^36,37^ and NV-centers in nanodiamonds,^38^ while SPSs based on III–V semiconductors offer longer operation lifetimes with barely any emission intermittency but require cryogenic temperatures.^39^ Other carbon-based SPSs operating at room temperature^21,40^ offer more stable emission, i.e. nearly nonblinking behavior for up to 1 h. This enhanced stability comes at the cost of brightness, as our C_60_ fullerene molecules are approximately ten times brighter than both graphene-based^21^ and carbon nanotube-based^40,41^ SPSs. The degradation of our SPSs is likely related to the optical excitation by our laser; therefore, using electrical excitation could be a potential strategy to mitigate it.^30^

The blinking behavior indicates the single photon emitting nature of our sources. For a more stringent demonstration of the operation of our source as an SPS, we characterize the antibunching behavior of the emitted light. We rely on the second-order autocorrelation function. It is calculated by plotting a histogram of the time delays between photon detection events recorded in different detectors of the HBT setup depicted in Figure 1a. A value of the normalized second-order autocorrelation function around zero time delays below 0.5, (*g* ^(2)^(τ = 0) < 0.5) indicates that the source behaves as a single-photon emitter.^42^

To obtain the second-order autocorrelation graph under continuous wavelength (CW) excitation, we use the 405 nm laser with an intensity of 0.891 kW/cm^2^, measured at the entrance of the objective, as shown in Figure 3b. The experimental points are correctly fitted by the function

1 $$g_{\mathrm{CW}}^{\left(2\right)}\left(\tau \right)=a\left(1-b\cdot e^{-\left|\tau -\tau_{0}\right|/\tau_{X}}\right)$$

where τ is the delay time between events. τ_0_ is the time offset between the two arms of the HBT setup and τ_*X*_ is the lifetime of the exciton. Then, the normalized second-order autocorrelation function reads,

2 $$g_{\mathrm{norm}}^{\left(2\right)}\left(\tau \right)=\frac{1}{a}\cdot g^{\left(2\right)}\left(\tau \right)$$

The function is normalized such that *g*_norm_^(2)^ → 1 for time delays much larger than the lifetime of the exciton. The value obtained for τ_*X*_ in Figure 3 b) is 4.700 ± 0.692 ns, while the value of the normalized *g* ^(2)^ function at τ = τ_0_ is 0.304 ± 0.024 ns. Notably, the value around time delays equal to zero is below 0.5, thus, the source under investigation behaves as a single-photon emitter.

**Figure 3:**
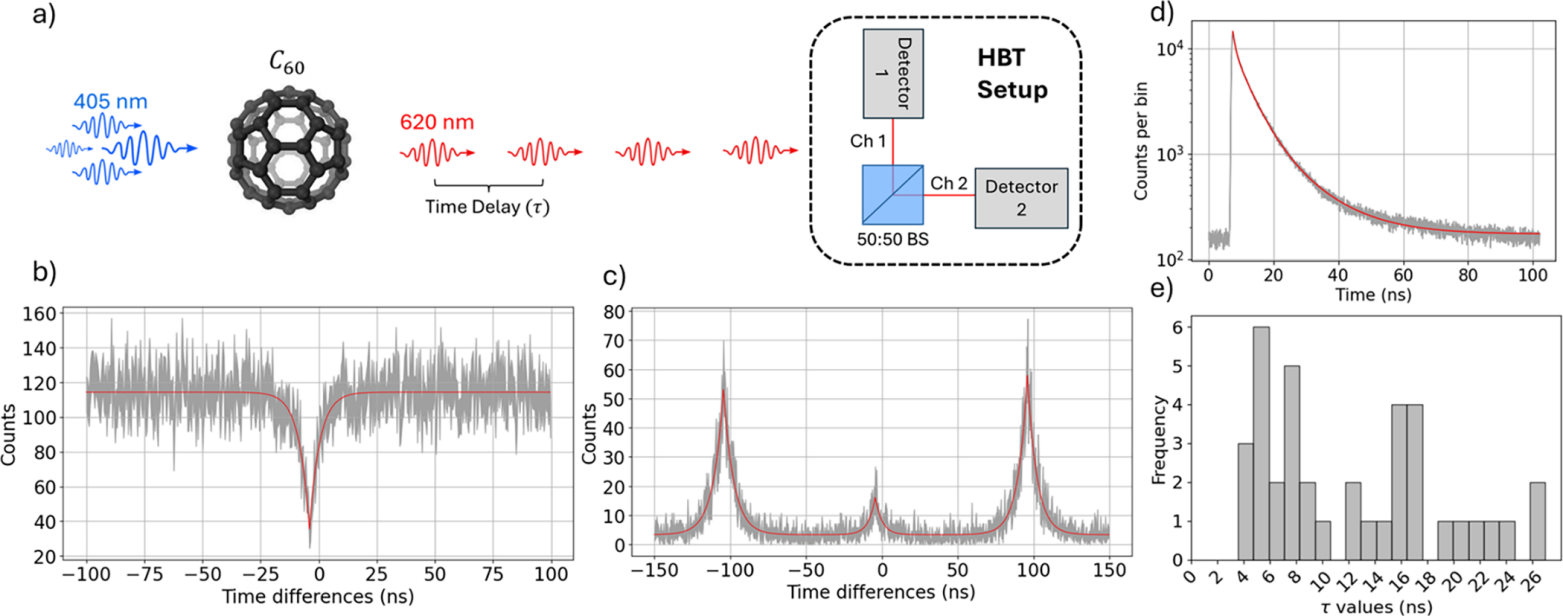
a) Scheme of the second-order autocorrelation measurement. We illustrate the process involving the excitation of a single C_60_ molecule with 405 nm laser light and the subsequent antibunched emission of light around 620 nm is directed toward the HBT setup for analyzing its emission statistics. b), c) second-order autocorrelation functions for the same SPS under CW excitation and PW excitation, respectively. In both graphs, the bin width is 500 ps. d) Lifetime histogram of the same SPS used for obtaining the CW and PW second-order autocorrelation measurements. e) Histogram representing the dispersion of lifetimes values obtained for different single photon sources. For the graphs b), c) and d), the values shown on the *y* axis correspond to the actual values along with their corresponding error bars.

Working under pulsed excitation allows us to achieve on-demand photon emission. In this mode, after each excitation pulse, the source emits one photon (or none, in case the excitation was unsuccessful or the source was ionized). Using a laser with pulse durations much shorter than the decay lifetime of our sources is crucial to avoid multiple excitations within a single laser pulse. Thus, with the same C_60_ molecule and changing to PW excitation with a repetition rate of 10 MHz and an intensity of 0.114 kW/cm^2^, we can obtain the second-order autocorrelation graph shown in Figure 3c. In this case, the experimental points are fitted with,

$$g_{\mathrm{PW}}^{\left(2\right)}\left(\tau \right)=a+b_{0}\cdot e^{-\left|\tau -\tau_{0}\right|/\tau_{X}}+\underset{n\neq 0}{\sum }b_{n}\cdot e^{-\left|\tau -\tau_{0}-n\cdot T\right|/\tau_{X}}\times (1-e^{-\left|\tau -\tau_{0}\right|/\tau_{X}})\cdot$$ 3

Where *b*_*i*_ is the height in number of counts of each of the different peaks and *T* is the time interval between pulses.^14^ We use the mean value of the height in counts of all the different peaks surrounding the peak at τ = τ_0_ to obtain the normalized second-order autocorrelation function

4 $$g_{\mathrm{norm}}^{\left(2\right)}\left(\tau \right)=\frac{1}{{\mathrm{b̅}}_{n\neq 0}}\cdot g^{\left(2\right)}\left(\tau \right)$$

Here *b̅*_*n*≠0_ is the mean value of all the *b*_*n*_ with *n* ≠ 0. The value of the normalized function at τ = τ_0_ is equal to 0.308 ± 0.024 ns, while the value for the lifetime of the exciton is 4.537 ± 0.655 ns. As in the case of CW illumination, the minimum value of the second-order autocorrelation function around time delays equal to zero is below 0.5, indicating the single-photon emission nature of the analyzed source.

Finally, we measure the decay lifetime of C_60_ molecules, directly linked to their emission rate, and, consequently, their potential use in high-speed quantum communications. To this end, we couple all the light emitted by our SPSs into a detector and record the arrival times of the detected counts. The time differences between each photon arrival and the preceding synchronization pulse of the pulsed laser is shown in Figure 3d. The data is well fitted by a multiexponential^43^

$$f\left(\tau \right)=A+\overset{3}{\underset{i=1}{\sum }}B_{i}\cdot e^{‐\frac{\left|\tau -\tau_{0}\right|}{\tau_{i}}}$$ 5

The fitted values of the different decay lifetimes are τ_1_ = 0.833 ± 0.015 ns, τ_2_ = 4.959 ± 0.095 ns and τ_3_ = 13.135 ± 0.450 ns. We interpret the first decay time as corresponding to the biexciton, the second to the exciton, and the third to a long-lived state. This suggests that the emission level scheme of our system involves a more complex dynamics than what a simple two-level model would imply. The averaged decay lifetime,

$$\tau_{\mathrm{avg}.}=\frac{\overset{3}{\underset{i=1}{\sum }}B_{i}\cdot \tau_{i}}{\overset{3}{\underset{i=1}{\sum }}B_{i}}$$ 6

gives, τ_avg*.*_ = 4.516 ± 0.079 ns.

Interestingly, we find different regimes in the measured exciton lifetime for different measurements: in some cases, we observe that they are on the order of around 4 ns, while in others, it extends to around 20 ns, as shown in Figure 3e. This variability in radiative decay times suggests the existence of different local environments or emissions pathways that affect the emission dynamics. Furthermore, decay lifetimes of around 4 ns are much shorter than those typically observed in other single photon sources, such as colloidal quantum dots or NV-centers under similar conditions and without any cavity coupling.^14,15,18,44,45^ Such short emission lifetimes result in higher emission rates, making these sources brighter, as more photons are emitted within a fixed time interval. These results are comparable to those of other carbon-based single photon sources, such as graphene,^21^ and significantly higher than those observed in carbon nanotubes.^22–24,40^

Fullerene C_60_ molecules can function as single-photon emitters at room temperature. Their emission can be triggered on demand using pulsed laser excitation. Blinking effects are significant in these sources, i.e. short and bright periods of emission. The distribution of the durations of the ON and OFF periods revealed that C_60_ molecules cannot be modeled with a three-level system. Despite the blinking, the source maintains a high degree of single-photon purity during the ON periods, making it a promising candidate for applications in quantum communication and quantum information processing.

Regarding the mechanisms that enable emission, our results suggest that 405 nm wavelength excitation plays a key role, triggering the photoassisted emission we observed, especially in highly concentrated samples. This process, caused by the photo-oxidation of fullerene molecules, increases their emissivity by breaking molecular symmetry through the addition of oxygen atoms to the structure. This change allows transitions that were previously forbidden.

While the exact nature of single-photon emission at the molecular level remains uncertain, we hypothesize that oxidation processes occurring either in individual molecules or within clusters may be responsible for this behavior. Such a mechanism could also explain variability observed in the exciton lifetimes across measurements.

Similarly to epitaxial quantum dots fabricated using III–V semiconductors,^8^ C_60_-based sources may be integrated into microchips that can be optically pumped by using electrically injected microlasers, offering the advantage of room-temperature operation. When compared to semiconductor quantum dots capable of operating at room temperature,^14,15^ our C_60_ molecules present shorter emission lifetimes, and consequently, higher emission rates.

Overall, the wide availability of C_60_, along with its low production cost and ease of preparation, marks a significant step toward the practical implementation of these molecules as single photon sources in quantum technologies.

## Supplementary Material



## Notes

Time-dependent photoluminescence spectra, Raman spectroscopy measurements, second-order autocorrelation and lifetime decay measurements compared with CdSe/ZnS core–shell colloidal quantum dots (PDF)
