# Supplementary material for: C60 Fullerene as an On-Demand Single Photon Source at Room Temperature
Source: Nano Lett. 2025 Oct 3;25(41):15048–54. doi: 10.1021/acs.nanolett.5c04007 (PMC12532277; doi:10.1021/acs.nanolett.5c04007)
Supplement: Supplementary file 1 [file nl5c04007_si_002.pdf]

Supporting Information: C_{60} fullerene as an on-demand single photon source at room temperature

Raul Lahoz Sanz,[*,†,‡] Lidia Lozano Martín,[‡,¶] Adrià Brú i Cortés,[§,‡] Sergi Hernández Márquez,[§,∥] Martí Duocastella,[¶,∥] Jose M. Gómez Cama,[§,‡,⊥] and Bruno Juliá-Díaz[*,†,‡]

†*Departament de Física Quàntica i Astrofísica, Facultat de Física, Universitat de Barcelona (QCommsUB group)*

‡*Institut de Ciències del Cosmos (ICCUB), Universitat de Barcelona (UB), c. Martí i Franqués, 1, 08028 Barcelona, Spain*

¶ *Department of Applied Physics, Universitat de Barcelona, C/Martí i Franquès 1, 08028, Barcelona, Spain*

§*Departament d'Enginyeria Electrònica i Biomèdica, Universitat de Barcelona (UB), c. Martí i Franqués, 1, 08028 Barcelona, Spain*

∥ *Institute of Nanoscience and Nanotechnology (IN2UB), Universitat de Barcelona (UB), 08028, Barcelona, Spain*

⊥*Institut d'Estudis Espacials de Catalunya (IEEC), Edifici RDIT, Campus UPC, 08860 Castelldefels (Barcelona), Spain*

E-mail: rlahozsanz@icc.ub.edu; brunojulia@ub.edu

Photoactivation of C_{60} molecules embedded in polystyrene

As reported in Zhang *et al.*,[S1] where C_{60} molecules embedded in polystyrene exhibit notable changes in their photoluminescent behavior upon prolonged laser irradiation, some graphs of how the photoluminescence spectra of our sources varies with time are showed in Fig. S1. In Zhang's article, their study demonstrated that extended exposure to laser light at specific wavelengths induces irreversible modifications in the C_{60} molecules, primarily due to photo-induced oxidation processes, leading to a substantial increase in fluorescence intensity and a blue shift in the emission spectrum.

In order to replicate their results, we have measured the spectra of the emitted light of our sample (the one prepared with the 1:100 dilution) when it is shined using large-field imaging with 1.5 mW of power. As the light is collected after the dichroic mirror, only wavelength above 567 nm are recorded in the spectra. Despite this, we can clearly observe how the intensity of the photoluminescence spectrum increases over time, reaching a maximum, and then begins to decrease again until we obtain a spectrum that is practically flat. This latter behavior may be due to a combination of the spectrum shifting towards higher-energy wavelengths, along with the fact that many of the molecules emitting light undergo photobleaching and cease to be emissive, which causes the intensity to drop.

In Fig. S1 a), we can see the enhancement of the photoluminescence spectra during the first 150 seconds. We can also see that at the beginning of the measurements we have two peaks, one at 700 nm and the other around 600 nm. Over time, we can observe that the peak initially found around 700 nm shifts toward higher energy wavelengths until it eventually disappears. In Fig. S1 b), we can see the different spectra taken from second 150 until the end of the measurement. Here, we observe how the photoluminescence spectrum begins to decrease almost to the point of vanishing. This process appears to be irreversible, as the shape of the spectra at the end of the measurement is no longer the same as what we observed at the beginning. We can see that the peak initially found around 700 nm never reappears. This suggests that the photoexcitation process is irreversible.

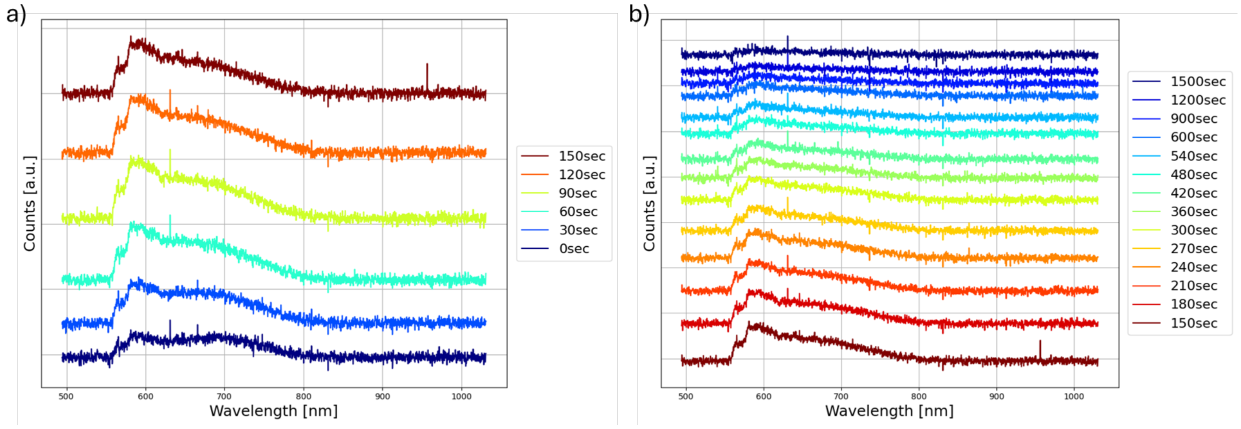

Figure S1: a) Photoluminescence spectra of the sample taken during the first 150 sec. b) Rest of the photoluminescence spectra taken up to a total time of 25 mins. The charts are arranged one on top of the other to aid visualization.

If we compare our work with that of Zhang, we notice that in our case, the characteristic waiting times to observe changes in the spectrum are much shorter, on the order of seconds in our work. This may be because we are using a more energetic laser to excite the samples, which could enhance the photoactivation effect, making it occur more rapidly.

Raman spectrometry

Raman spectroscopy measurements were conducted using an excitation wavelength of 532 nm, a 50× objective lens, and a maximum laser power of 6 mW (spectrometer LabRam HR 800, HORIBA). The system provided a spectral resolution of approximately 1 cm^{-1}. Acquisition times were varied up to 120 s, with up to 10 accumulations per spectrum. During the measurements using the 532 nm laser, the sample exhibited significant fluorescence background and was prone to photodamage at higher laser powers, therefore low powers of less than 1% were used to minimize these effects. All the measured peaks in our experiment are summarized in table S1 and shown in Fig. S2. The primary objective of the Raman shift analysis was to confirm that the sample was made of pristine C_{60} and to detect the presence of other carbon structures, such as C_{70} or polymerized C_{60}.

In pristine C_{60}, carbon atoms are arranged at the vertices of fused pentagonal and hexagonal rings, forming a highly symmetric, spherical structure.[S2] C_{60} possesses 174 vibrational modes, of which 10 are Raman-active, corresponding to two A_g modes and eight H_g modes.[S3] Among these, the $A_g(2)$ mode exhibits the highest Raman intensity, associated with the "pentagonal pinch" vibration. This mode is particularly sensitive to molecular symmetry and environmental changes, with its frequency ranging from approximately 1470 cm^{-1} to 1459 cm^{-1} depending on external factors .[S3] As observed in Fig. S2, the $A_g(2)$ peak appears at 1467 cm^{-1}. The presence of a secondary left peak at 1458 cm^{-1}, named in this work as $A_g(2)*$, is indicative of polymer formation, likely initiated by exposure to ambient light, as well as the formation of C_{60} dimers or $C_{60}O_2$ complexes.[S3,S4]

Notably, the $H_g(8)$ frequency measured in our analysis in Fig. S2 appears consistent with reported values for C_{70},[S2,S5,S6] suggesting the possible presence of higher fullerene species. The source of smaller peaks highlighted in green in Fig. S2 is undetermined, as they don't match with reported C_{70} spectra nor higher forms of C_{60}. They could indicate oxidation, degradation of the sample or disorder modes. The broad peak in Fig. S2 at 970 cm^{-1} comes from the measuring system. The low intensity of the H_g modes and the absence of $H_g(5)$ in Fig. S2 could be indicative of the presence of oxidation on the C_{60} molecules.[S7]

Although the samples exhibit minor signs of degradation or oxidation –likely due to air exposure during measurements– the Raman spectra are consistent with those reported for pristine C_{60}, supporting the conclusion that the samples are primarily composed of unmodified fullerene molecules.

Table S1: C_{60} Raman shift (cm^{-1}) at 532 nm excitation wavelength

Symmetry	$A_g(1)$	$A_g(1)*$	$A_g(2)$	$A_g(2)*$	$H_g(1)$	$H_g(2)$	$H_g(3)$	$H_g(4)$	$H_g(5)$	$H_g(6)$	$H_g(7)$	$H_g(8)$
532 nm excitation	495	–	1467	1458	269	430	706	770	–	1243	1425	1565

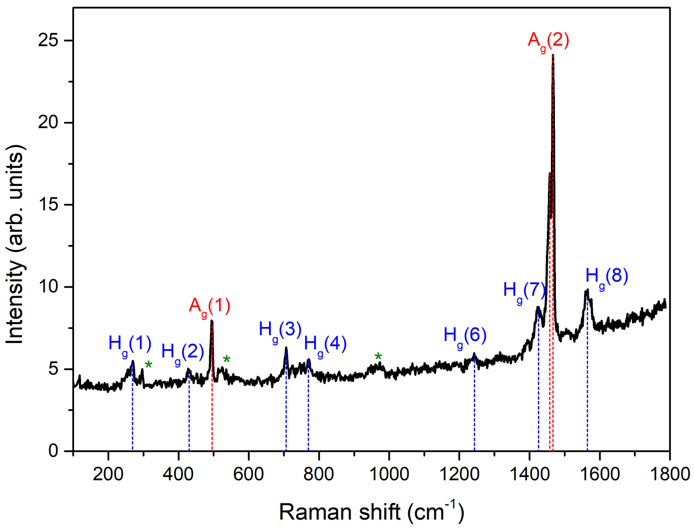

Figure S2: C_{60} Raman shift at 1% of power, acquired at 532 nm wavelength.

Comparison C_{60} vs. CQDs

To highlight the main differences between colloidal quantum dots (CQDs) and C_{60} molecules, we present a comparison of the typical plots of both the second-order autocorrelation function and the emission decay lifetime obtained for each type of single photon source.

For the preparation of the colloidal quantum dot sample, we used a 1:1,000,000 dilution of CdSe/ZnS core-shell colloidal quantum dots (*900219, Sigma-Aldrich*) in dry toluene with 5% dissolved polystyrene. Once this solution was prepared, a drop was spin-coated onto a gold-coated slide at a speed of 4000 rpm for 1 minute.

In Fig. S3 a) we find a comparison between the second-order autocorrelation function under CW excitation using a sample of CQDs (red) and C_{60} (blue). We find that the blue graph has a lower decay lifetime, as the dip is more pronounced. Regarding the value of the normalized second-order autocorrelation function and the decay lifetime, using eq. (1) and eq. (2), we obtain that, for the CQD, the values are

$$g^{(2)}_{norm.}(\tau = \tau_0) = 0.186 \pm 0.007, \text{ and } \tau_X = 22.35 \pm 0.37 \text{ ns},$$

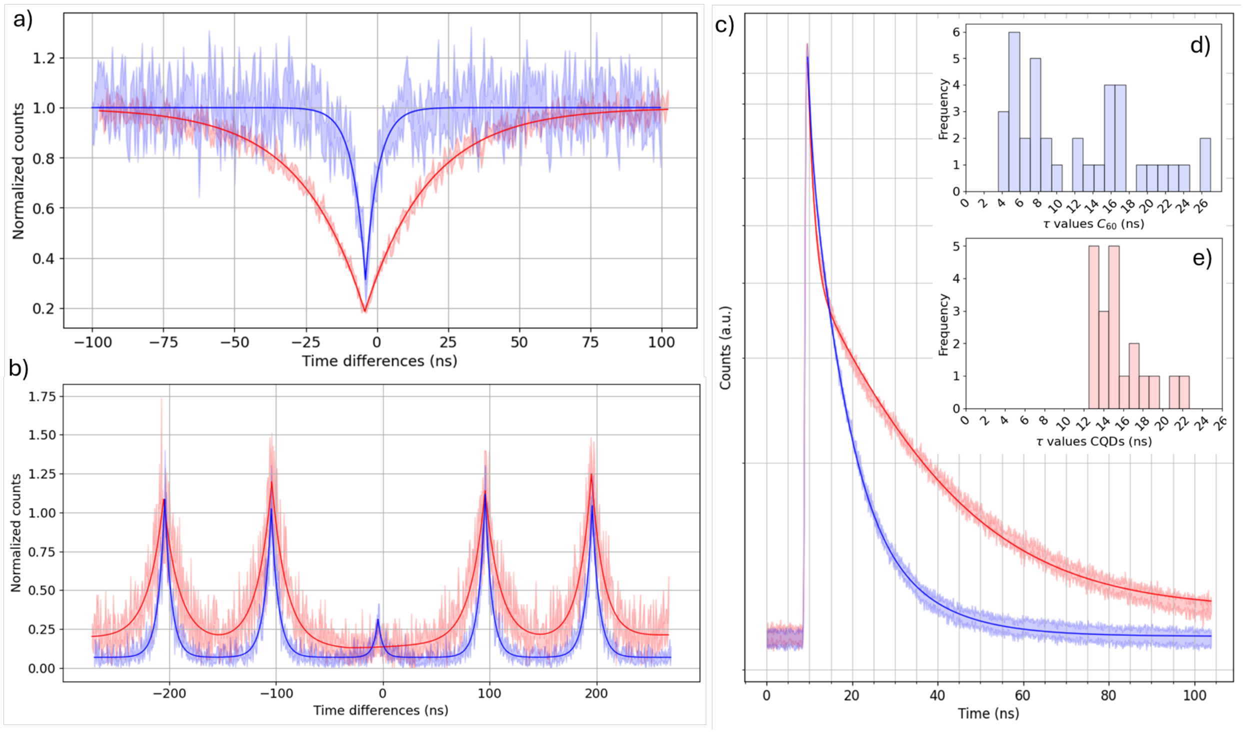

Figure S3: C_{60} (blue) vs CQD (red) comparison. a) b) Second-order autocorrelation functions comparison under CW excitation and PW excitation, respectively. In all graphs, the bin width is 500ps. c) Lifetime comparison, where counts on the y-axis are depicted in logarithmic scale. The fit function was adjusted to a bi-exponential decay. d) Histogram of the different decay lifetimes of the C_{60} SPS. e) Histogram of the different decay lifetimes of the CdSe/ZnS core-shell colloidal quantum dots. It is worth mentioning that we used the same C_{60} single-photon source for all the blue measurements. For the red measurements, the CW second-order autocorrelation function and the decay lifetime were obtained using the same CQD, while the PW second-order autocorrelation function was measured using a different one.

while for the C_{60} the values are

$$g^{(2)}_{norm.}(\tau = \tau_0) = 0.312 \pm 0.039, \text{ and } \tau_X = 4.44 \pm 0.37 \text{ ns}.$$

On the other hand, for PW excitation, we found the comparison in Fig. S3 b). Now, using the equations (3) and (4), we obtain that, for the CQD, the values are

$$g^{(2)}_{norm.}(\tau = \tau_0) = 0.304 \pm 0.024, \text{ and } \tau_X = 15.316 \pm 0.736 \text{ ns},$$

while for the C_{60} the values are

$$g^{(2)}_{norm.}(\tau = \tau_0) = 0.304 \pm 0.024, \text{ and } \tau_X = 4.700 \pm 0.692 \text{ ns}.$$

For the C_{60} (blue) lifetime graph showed in Fig. S3 c) we have fitted the data to a multi-exponential function like in the eq. (5). Obtaining the same results, $\tau_1 = 0.833 \pm 0.015$ ns, $\tau_2 = 4.959 \pm 0.095$ ns and $\tau_3 = 13.135 \pm 0.450$ ns. These results leads us to an averaged decay lifetime of $\tau_{avg.} = 4.516 \pm 0.079$ ns, by using the eq. (6).

On the other hand, for the CQD (red) lifetime graph showed in Fig. S3 c), we have fitted the values to a biexponential decay,[S8] obtaining $\tau_1 = 24.164 \pm 0.103$ ns and $\tau_2 = 1.334 \pm 0.009$ ns. Here, τ_1 corresponds to the decay lifetime of the exciton while τ_2 corresponds to the decay lifetime of the biexciton.

Finally, the histograms presented in Fig. S3 d) and e) show the dispersion of the different decay lifetimes observed in both C_{60} and CQDs. We can clearly see that C_{60}-based SPS have a broad distribution of lifetimes, suggesting different emission environments and greater ease of being disturbed by the environment. While the CQD lifetime distribution is mainly concentrated in the region between 13 ns and 19 ns, the C_{60} lifetime distribution ranges from 3 ns to 27 ns.

References

(S1) Zhang, C.; Xiao, X.; Ge, W.; Loy, M.; Dazhi, W.; Qijin, Z.; Jian, Z. Photoluminescence study of C60 doped polystyrene. *Applied physics letters* **1996**, *68*, 943–945.

(S2) Bethune, D. S.; Meijer, G.; Tang, W. C.; Rosen, H. J. The vibrational Raman spectra of purified solid films of C60 and C70. *Chemical Physics Letters* **1990**, *174*, 219–222.

(S3) Khinevich, N.; Girel, K.; Bandarenka, H.; Salo, V.; Mosunov, A. Surface enhanced

Raman spectroscopy of fullerene C60 drop-deposited on the silvered porous silicon. *Journal of Physics: Conference Series* **2017**, *917*, 062052.

(S4) Dorner-Reisel, A.; Ritter, U.; Moje, J.; Freiberger, E.; Scharff, P. Effect of fullerene C60 thermal and tribomechanical loading on Raman signals. *Diamond and Related Materials* **2022**, *126*, 109036.

(S5) Sun, G.; Kertesz, M. Vibrational Raman Spectra of C70 and C706- Studied by Density Functional Theory. *The Journal of Physical Chemistry A* **2002**, *106*, 6381–6386.

(S6) Schettino, V.; Pagliai, M.; Cardini, G. The Infrared and Raman Spectra of Fullerene C70. DFT Calculations and Correlation with C60. *The Journal of Physical Chemistry A* **2002**, *106*, 1815–1823.

(S7) Zygouri, P.; Spyrou, K.; Mitsari, E.; Barrio, M.; Macovez, R.; Patila, M.; Stamatis, H.; Verginadis, I.; Velalopoulou, A.; Evangelou, A.; Sideratou, Z.; Gournis, D.; Rudolf, P. A facile approach to hydrophilic oxidized fullerenes and their derivatives as cytotoxic agents and supports for nanobiocatalytic systems. *Scientific Reports* **2020**, *10*, 8244.

(S8) Ihara, T.; Miki, S.; Yamada, T.; Kaji, T.; Otomo, A.; Hosako, I.; Terai, H. Superior properties in room-temperature colloidal-dot quantum emitters revealed by ultralow-dark-count detections of temporally-purified single photons. *Scientific Reports* **2019**, *9*, 15941.
